# TRP Channels as Lower Urinary Tract Sensory Targets

**DOI:** 10.3390/medsci7050067

**Published:** 2019-05-22

**Authors:** Karl-Erik Andersson

**Affiliations:** 1 Institute for Regenerative Medicine, Wake Forest University School of Medicine, Winston Salem, NC 27101, USA; karl-erik.andersson@med.lu.se; Tel.: +46705522722; 2 Institute of Laboratory Medicine, Lund University, 223 62 Lund, Sweden

**Keywords:** lower urinary tract, bladder, urethra, TRPV1, TRPV2, TRPV4, TRPM8 and TRPA1

## Abstract

Several members of the transient receptor potential (TRP) superfamily, including TRPV1, TRPV2, TRPV4, TRM4, TRPM8 and TRPA1, are expressed in the lower urinary tract (LUT), not only in neuronal fibers innervating the bladder and urethra, but also in the urothelial and muscular layers of the bladder and urethral walls. In the LUT, TRP channels are mainly involved in nociception and mechanosensory transduction. Animal studies have suggested the therapeutic potential of several TRP channels for the treatment of both bladder over- and underactivity and bladder pain disorders,; however translation of this finding to clinical application has been slow and the involvement of these channels in normal human bladder function, and in various pathologic states have not been established. The development of selective TRP channel agonists and antagonists is ongoing and the use of such agents can be expected to offer new and important information concerning both normal physiological functions and possible therapeutic applications.

## 1. Introduction

The transient receptor potential (TRP) superfamily comprises a diverse group of cation channels involved in many cellular functions, both in the normal state and in different pathophysiologies [1]. Modulating TRP channel activity provides an important way to regulate membrane excitability and intracellular calcium levels. This is reflected by the fact that small molecule compounds modulating TRP vanilloid 1 (TRPV1), TRPV3, TRPV4, TRP ankyrin 1 (TRPA1), and TRP melastatin 8 (TRPM8) have all entered clinical trials for a variety of diseases [2]; however, except for capsaicin and resiniferatoxin, none of the newer agents seem to have been tested in disorders affecting the lower urinary tract (LUT). The occurrence of capsaicin-sensitive structures in the human bladder was functionally demonstrated in 1989 [3] and this spurred interest in the therapeutic potential of TRP channels for the treatment of LUT disorders. The subject has been discussed in many reviews [1,4,5,6,7,8,9,10,11] which have concluded that several TRP channels are expressed in the bladder, acting as sensors of stretch and/or chemical irritation. TRP channels, including TRPV1, TRPV2, TRPV4, TRM4, TRPM8 and TRPA1, are highly expressed in, but not restricted to, primary afferent neurons, but can also be found in the urothelium [12], some interstitial cells and detrusor muscle [7,11,13]. However, how these individual channels influence normal and pathological LUT function remains to be established.

The aim of this review is to summarize the background and provide an update on the role of TRP channels in bladder function/dysfunction, and of their possible therapeutic potential

## 2. TRPV1 and Bladder Function

The expression pattern and properties of TRPV1 channels have been well described. It is a non-selective cationic channel with high Ca^2+^ permeability, allowing the passage of cations, mainly Ca^2+^, and it is activated by vanilloids, noxious heat and low pH [14,15]. It is the best-characterized member of the TRPV subfamily (TRPV1–6) and abundant information on its morphology and function in animal models, and to some extent on the clinical translation of its manipulation, is available [1,16,17]. Despite this, the role of TRPV1 in normal human bladder function is still controversial. However, its role in the pathophysiology and treatment of particularly neurogenic detrusor overactivity (NDO) has been well demonstrated [5].

TRPV1 expression has been observed in neuronal and non-neuronal human and rat LUT tissues including the urothelium, suburothelial nerve plexus, detrusor smooth muscle, interstitial cells, and afferent neurons. There is evidence for TRPV1 expression in small diameter bladder afferent fibres in close proximity to the urothelium and in bladder sensory neurons in the dorsal root ganglia (DRG). However, the expression, particularly in the urothelium, has been controversial [11].

Daly et al. [18] compared single-unit bladder nerve recordings in TRPV1 knockout mice (*trpv1^-/-^*) and their TRPV1 littermates. They found that low-threshold neuronal responses were attenuated whereas high-threshold sensitivity was unchanged, and further suggested that the neuronal TRPV1 channels in the suburothelium were needed for normal excitability of low-threshold bladder fibers. In cystometrogram recordings *trpv1^-/-^* mice showed an increase in the frequency of non-voiding contractions but a regular pattern of voiding contractions. In voiding behavior studies, these mice showed enhanced intermicturition spotting, whereas normal micturitions seemed to be unaffected [4]. These findings were confirmed by Yoshiyama et al. [19] using a dual analysis of voiding behavior and reflex micturition in cystometric studies.

In urethane anesthetized *trpv1^-/-^* mice an increase in mean bladder capacity and a reduction in spinal cord c-fos induction in response to bladder distension was demonstrated [20], suggesting that TRPV1-mediated mechanisms are responsible for setting the micturition threshold under anesthesia. In contrast, conscious *trpv1^-/-^* mice showed an unaffected micturition frequency, suggesting that under voluntary conditions non-TRPV1-mediated mechanisms set the threshold.

In studies of patients with NDO, the increased immunoreactivity of PGP9.5 (nerve stain) and TRPV1 were found in the suburothelium and basal layers of the urothelium compared to control patients. The TRPV1 immunoreactivity was significantly decreased in NDO patients clinically responding to intravesical instillations of resiniferatoxin (RTX), suggesting a role for TRPV1 in the pathophysiology of NDO [21,22,23]. However, the effects of vanilloids (capsaicin, RTX) on urothelial TRPV1 indicated that vanilloid actions were more complex than simple C fiber desensitization.

Since both TRPV1 and P2X receptors are present on bladder sensory nerve fibres and have been implicated in mechanosensation during bladder filling, Grundy et al. [24], using *trpv1^-/-^* mice, determined possible interactions between these receptors in modulating afferent nerve activity. They found that TRPV1 modulates P2X mediated afferent responses and suggested this mechanism to explain the decrease in sensory symptoms observed following RTX and capsaicin used for treatment LUT symptoms. Zhang et al. [25] studied the expression of TRPV1 in the urothelium of 21 female patients with overactive bladder (OAB). They found that the expression was significantly higher in the patients than in nine healthy controls. The high expression of TRPV1 in the urothelium of the patients was closely correlated to OAB occurrence. Zhang et al. [25] also found that urodynamic parameters such as maximum flow rate (Qmax), first desire volume, strong desire volume, maximum cystometric capacity and bladder compliance were lower in OAB patients than in healthy females. This is in line with previous studies. Liu et al. [26], investigating patients with OAB symptoms without demonstrable DO, but an early first sensation during bladder filling due to sensory discomfort (sensory urgency), found an increased TRPV1 mRNA expression in the trigonal mucosa. The TRPV1 expression levels in the trigone were inversely correlated to the volume at first sensation during bladder filling. In contrast, patients with idiopathic DO (IDO) there were no changes in TRPV1 expression levels, suggesting a distinct molecular basis between sensory urgency and IDO [26].

Exposure at an early age to various agents affecting TRPV1 channels may predispose a patient to the later development of bladder dysfunction. Park et al. [27] subjected ten-day-old rat pups to bladder sensitization via an intravesical infusion of acetic acid in saline with or without prior bladder desensitization with capsaicin. They showed that the stimulation, which did not cause significant inflammation, could induce bladder sensitization and that TRPV1 played a role in inducing and maintaining bladder sensitization persisted in adult rats.

Not only neonatal sensitization, but also social stress may cause profound urinary bladder dysfunction in children that often continues into adulthood. Thus, social stress can ultimately lead to the development of OAB by the induction of TRPV1-dependent afferent nerve activity [28]. Mingin et al. [28] exposed six-week-old male C57BL/6 mice via barrier cage, to a C57BL/6 retired breeder aggressor mouse, and performed conscious cystometry with and without intravesical infusion of the TRPV1 inhibitor capsazepine, and measured pressure-volume relationships and afferent nerve activity during bladder filling using an ex vivo bladder model. Stress leads to a decrease in intermicturition interval and voided volume in vivo, which was restored by capsazepine. Ex vivo studies demonstrated that at low pressures, bladder compliance and afferent activity were elevated in stressed bladders compared with unstressed bladders. It was concluded that social stress could induce TRPV1- dependent afferent nerve activity, ultimately leading to the development of OAB. Further study [29] demonstrated that TRPV1 channels may also play a role in the development of bladder decompensation and underactivity in an intensified model of social stress, suggesting that TRPV1 channels can be an interesting target to prevent the development of stress-induced bladder dysfunction in children.

TRPV1-IR (immunoreactive) nerves seem to be distributed to all parts of the urogenital tract, including the urethra, and capsaicin seems to have effects on both urethral smooth and striated muscles [4]. It has been speculated that DO may be initiated from the urethra [30], and in females, a rapid pattern of urethral pressure variation (“unstable urethra”) seems to be closely associated with DO [30,31,32,33,34]. This raises the question of whether the TRPV1 channel is involved in urethral functions that can be linked to DO/OAB.

## 3. TRPV2 Channels and Bladder Function

TRPV2 is a nonselective cation channel with high Ca^2+^ permeability [35,36]. It may act as a mechanosensor, thermosensor and lipid sensor [36]. In vascular smooth muscle cells TRPV2 was shown to be a stretch-activated channel able to increase stretch-induced [Ca^2+^]_i_ [37]. As a heat sensor TRPV2 has a temperature threshold of 50–52 °C and it is activated by agonists such as 2-aminoethoxydiphenyl borate and D9-tetrahydrocannabinol (THC) [35].

In rat urinary bladder TRPV2 mRNA expression was found in urothelial and smooth muscle cells [20]. The channel was also functionally demonstrated in mouse urothelial cells [12,38]. In the human bladder Ost et al. [39] found immunostaining for TRPV2 in small nerve fibers, suburothelial cells and smooth muscle cells, but the specificity of the antibodies was uncertain [4]. Caprodossi et al. [40] found TRPV2 expression not only in normal human urothelial cells and bladder tissue specimens, but also in sensory DRG neurons.

The functional significance of the TRPV2 channel in the LUT is still unclear. However, it has been suggested that TRPV2 could have a pivotal role in bladder cancer development [41,42,43].

## 4. TRPV4 Channels and Bladder Function

TRPV4 is a broadly expressed, polymodally gated ion channel that plays an important role in many physiological and pathophysiological processes [44,45,46]. It is opened in response to heat, mechanical stimuli, hypo-osmolarity and arachidonic acid metabolites [47]. TRPV4 has been suggested to be an important urothelial mechanosensor for bladder distension [7,48].

TRPV4 channels are expressed in rat and mouse urothelial and detrusor muscle cells [49,50,51]. In rat urothelial cells activation of TRPV4 induces significant increases in [Ca^2+^]_i_, leading to ATP release [52].

Using the TRPV4 agonist, GSK1016790A, Isogai et al. [50] investigated the role of TRPV4 channels in regulating the contractility of detrusor smooth muscle and muscularis mucosae of the guinea pig bladder. The agonist evoked a sustained contraction in both tissues, abolished spontaneous Ca^2+^ transients, increased basal Ca^2+^ and abolished spontaneous phasic contractions. The large conductance Ca^2+^-activated K^+^ (BK) channel blocker, iberiotoxin, restored the spontaneous contractions, and it was concluded that Ca^2+^ influx through TRPV4 activated BK channels to suppress spontaneous contractions. Thus, a functional coupling of TRPV4 with BK channels may act as a self-limiting mechanism for bladder contractility during its storage phase. Lee et al. [53] tested the hypothesis that TRPV4 channels are expressed primarily by PDGFRα+ cells in detrusor muscle, and that Ca^2+^ entry via TRPV4 channels is linked to activation of small Ca^2+^- activated K^+^ (SK) channels. They found that enhanced Ca^2+^ influx via TRPV4 channels during bladder filling increased the open probability of SK channels to dampen the excitability of detrusor muscles. Thus, TRPV4-mediated Ca^2+^-influx may activate both BK- (detrusor) and SK (PDGFRα+) channels, leading to decreased bladder contraction and facilitation of the storage phase.

The deletion of TRPV4 had consequences for the micturition pattern. *TRPV4*^-/-^ mice showed significantly more intermicturition spotting than wild-type animals, whereas the individual voidings appeared normal. Cystometry in conscious mice showed increased intermicturition intervals and increased numbers of non-micturition contractions [54]. In vitro the amplitude of spontaneous contractions in bladder strips was significantly reduced, and there was a decreased intravesical stretch-evoked ATP release in isolated whole bladders [54]. These observations suggest that TRPV4 plays a critical role in urothelium-mediated transduction of intravesical mechanical pressure, supporting the results of Mochizuki et al. [55].

Thus, the mechanical stimulus-dependent activation of TRPV4 in the urothelium could be a key event for ATP signaling in the micturition reflex pathway.

However, TRPV4 channels may participate in a mechanosensory pathway in the detrusor. TRPV4 receptors are also located to detrusor muscle and TRPV4 activation with GSK1016790A contracted normal mouse bladders in vitro, both in the presence and absence of the urothelium; this effect could not be found in bladders from *TRPV4*^-/-^ [56]. The amplitude of the spontaneous contractions in bladder strips from *TRPV4*^-/-^ mice was significantly reduced compared to controls [54].

In vivo, the direct infusion of GSK1016790A into the bladders of normal mice induced DO but had no effect in *TRPV4*^-/-^ mice.

Taken together, the observations suggest that the TRPV4 channels in the bladder urothelium and detrusor smooth muscle together play important roles in urinary bladder function. Additionally, TRPV4 channels in the urethra may be able to detect urine flow in the urethra, hereby activating the urethra-to-bladder reflex, promoting bladder emptying [4,57,58]. The mechanisms responsible for bladder-to-bladder and urethra-to-bladder reflexes are still poorly understood. In the awake ewe, urethra-to-bladder reflexes were stimulated by urethral infusion saline at body temperature, but not at temperatures below the physiological rate [58]. TRPV4 channels are activated by shear stress at body temperature and involvement in the mediation of the urethra-to-bladder reflex cannot be excluded [4].

TRPV4 channels may thus be involved in the mechanosensory pathway in urothelial cells with consequent release of ATP, and in direct stimulation of detrusor muscle. However, this may not be the only ways for initiation of the micturition reflex. The findings of TRPV4 in sensory neurons [59,60] suggest that the TRPV4 channels on bladder sensory terminals may mechanically be gated by bladder distension without any release of chemical mediators from the urothelium.

## 5. TRPA1 Channels and Bladder Function

TRPA1 is the only mammalian member of the Ankyrin TRP subfamily [61,62,63]. It is known to be expressed in a subset of capsaicin-sensitive primary sensory neurons where it acts as a polymodal sensor for diverse physical and chemical stimuli of extracellular or intracellular origin. It is characterized by a marked promiscuity of activating agents towards painful or potentially harming stimuli, including allylisothiocyanate (AI), cinnamonaldehyde (CA) [64,65,66], hydrogen sulfide (H2S), menthol and formalin [64,65,66,67,68,69,70,71].

In conscious rats, Streng et al. [67] performed cystometric investigations in conscious animals subjected to intravesical administration of known TRPA1 activators such as sodium hydrogen sulfide (NaHS, donor of H_2_S), AI and CA. Fluorometric calcium imaging was used to study the effect of NaHS on human and mouse TRPA1 expressed in CHO cells. Allylisothiocyanate increased micturition frequency and reduced voiding volume. Cinnamonaldehyde and NaHS produced similar changes in urodynamic parameters after disruption of the urothelial barrier with protamine sulfate. NaHS also induced calcium responses in TRPA1-expressing CHO cells, but not in untransfected cells. The finding that intravesical TRPA1 activators initiate DO indicated that TRPA1 may have a role in sensory transduction in this organ. The study also highlighted H_2_S as a TRPA1 activator potentially involved in inflammatory bladder disease. Supporting this view, Nicholas et al. [69] studying the mechanism of action of H_2_O_2_ on the major types of bladder afferents found that H_2_O_2_ was more potent in activating capsaicin-sensitive high threshold afferents than low threshold stretch-sensitive afferents and that H_2_O_2_ probably acted mostly via TRPA1. They suggested that the TRPA1 channels located on capsaicin-sensitive afferent fibres are probable targets of reactive oxygen species (ROS) released during oxidative stress, supporting the view of Andersson et al. [72].

In the human urethra, TRPA1 channels have been demonstrated on urothelial cells and on C-fiber afferents in the lamina propria and detrusor muscle [73]. Many TRPA1-IR nerves also expressed immunoreactivity for TRPV1. In urethral strip preparations, TRPA1-agonists had no contractile effect but after precontraction with phenylephrine, AI, CA, and NaHS caused concentration-dependent relaxations of the preparations. These relaxations were enhanced by capsaicin, were negatively coupled via cannabinoid receptor activation, and involved cyclooxygenase products [74]. The potential role urothelial TRPA1 channels in the regulation of normal human urethral smooth muscle tone is unclear, but this does not exclude a role in the initiation of afferent activity normally and in disease states [74]. Since the bladder and urethra work in concert, it may be speculated that TRPA1 together with TRPV4 channels may be involved in the urethra-to-bladder reflex.

## 6. TRPM4 and Bladder Function

The TRPM4 channel is a monovalent cation-selective channel activated by an increase of intracellular Ca^2+^. Upon activation, it allows Na^+^ entry into the cell but is completely impermeable to Ca^2+^ [75]. TRPM4 is implicated in the regulation of many cellular processes e.g., the immune response, insulin secretion, and bladder function (Cho et al., 2015). It is widely expressed in the body [76,77] including rat, guinea pig and human bladder urothelium and detrusor smooth muscle [12,78,79,80].

In isolated guinea-pig bladder smooth muscle strips, 9-phenanthrol, a selective inhibitor of the TRPM4 channel, reduced spontaneous contractions as well as contractions elicited by carbachol, KCl, and nerve stimulation [78,79,81]. It was also shown that 9-phenanthrol significantly reduced the intracellular Ca^2+^ levels, attenuated transient spontaneous inward currents and produced a hyperpolarizing shift in membrane potential, indicating that TRPM4 is active at rest, regulates detrusor cell excitability and contributes to contractile activity.

Alom et al. [82] investigated the involvement of TRPM4 channels in cholinergic contractile responses in detrusor smooth muscle preparations in mice. They found that 9-phenanthrol significantly inhibited carbachol-induced contractions but did not inhibit contractions due to intracellular Ca^2+^ release evoked by the drug, suggesting that the inhibitory effect was primarily due to the inhibition of the membrane depolarization process incurred by TRPM4 channels. The authors concluded that TRPM4 channels play a significant role in cholinergic signaling in detrusor smooth muscles.

These studies have suggested that TRPM4 could be a potential therapeutic target for detrusor overactivity. In line with this, Kullmann et al. [83] investigating the potential role of TRPM4 in detrusor overactivity following spinal cord transection (SCT) in mice, found that TRPM4 was upregulated in the urothelium and detrusor smooth muscle after the lesion. The spontaneous contractile activity of detrusor muscle strips in both spinally intact and STC mice was significantly reduced.

## 7. TRPM8 and Bladder Function

TRPM8 is a cool receptor expressed in the urothelium and suburothelial sensory fibers [84]. It is a Ca^2+^ permeable non-selective cation channel directly activated by cold temperatures and chemical agonists such as menthol [85,86].

In the LUT, TRPM8 is a well-established sensor of environmental cold temperatures. It is known that in healthy humans, low environmental temperature elicits more frequent urination, and that cold weather aggravates urgency symptoms in OAB patients [87]. As pointed out by Uvin et al. [88] it is highly unlikely that under normal environmental conditions, the temperature inside the bladder wall drops sufficiently to activate such cold-sensitive fibers. In conscious rats, a sudden drop of environmental temperature may change micturition patterns, and it has been suggested that these changes are mediated, at least in part, through RTX-sensitive (C-fiber) nervous pathways [87,88,89]. Interestingly, when menthol was applied to the skin from the leg and back in conscious rats, DO was induced [80]. TRPM8 was found to be expressed in the skin, and it was speculated that menthol, via stimulation of these receptors, was able to initiate that activity [87,89]. Uvin et al. [88] applied innocuously cold stimuli to different parts of the skin and found that this induced rapid bladder contractions and voids in anesthetized mice and rats. The responses were strongly attenuated in *Trpm8*^-/-^ mice and in rats treated with the TRPM8 antagonist AMTB, and it was suggested that the pharmacological inhibition of TRPM8 may be useful for treating acute cold-induced urgency symptoms in patients.

Based on the positive correlation between the density of TRPM8 channels in the bladder mucosa and voiding frequency in IDO, and the increased TRPM8 expression in bladder pain patients, it was suggested that these channels were involved in the symptomatology and pathophysiology of these disorders [81,82]. TRPM8 channels have also been implicated in the bladder cooling reflex (BCR) in humans [90,91] and animals [92,93]. Mukerji et al. [90,91] studied the BCR in patients with IDO and NDO. In both conditions a BCR could be elicited (IDO 6/22: 27%; NDO 4/4: 100%). The authors suggested that the BCR in DO reflected a loss of central inhibition of the micturition reflex.

Tsukimi et al. [94] found that the BCR can be elicited if guinea-pigs were pretreated with intravesical menthol. Since the reflex was sensitive to capsaicin treatment it was suggested to be mediated via TRPM8 receptors on C-fibres. Nomoto et al. [95] evaluated the effect of intravesical menthol in conscious rats to find a facilitation of the micturition reflex. However, the reflex was not affected by capsaicin pretreatment, and the authors suggested that menthol could act on capsaicin-resistant afferents, hypothetically via TPRM8 receptors in the urothelium and on suburothelial nerve endings. If species differences or mode of capsaicin pre-treatment (94: intravesical; 95: systemic) contributed to the difference in results is unclear. Du et al. [96] questioned the role of urothelial TRPM8 in human bladder sensory function, finding extremely low expression of TRPM8 mRNA in the bladder mucosa compared to the expression in the prostate. They also found that benign prostatic hyperplasia (BPH) or bladder outflow obstruction (BOO) did not significantly affect the expression of TRPM8, and further suggested that TRPM8 may play an essential role in the survival and proliferation of prostate epithelial cells, as suggested by Zhang and Barritt [97]. TRPM8 is highly expressed in the prostate [98] and there is growing evidence that TRPM8 should be studied within the frame of carcinogenesis, especially in the prostate [99].

## 8. TRP Channel Agonists and Antagonists

A considerable number of TRP channel ligands have been described acting as exogenous and endogenous channel agonists or antagonists [100]. TRP channel agonists/antagonist may be useful for elucidation of physiological functions and mechanisms involved in several LUT conditions. Many TRP agonists/antagonist have been tested pre-clinically both on normal LUT function and in various models of disease. Several potent, small-molecule TRPV1, TRPV3 and TRPA1 antagonists have already entered clinical trials as novel analgesic agents [100], and the development of TRP-selective agonists and antagonists is ongoing.

### 8.1. TRP Agonists

As mentioned, the neuronal TRPV1 channel may have pathophysiological roles contributing to OAB and pain [101]. The only TRP agonists that have been used clinically for LUT disorders are the toxins capsaicin and RTX, and their application has been extensively reviewed elsewhere [102,103,104,105,106,107]. These toxins are considered to act by eventually desensitizing the TRPV1 channel.

The effect profile of TRPV4 stimulation seems attractive for detrusor underactivity (DU) treatment. Deruyver et al. [108] administered the TRPV4 agonist GSK1016790A intravesically in a pelvic nerve injury rat model for DU. They found that the drug increased voiding frequency and reduced postvoid residual in wild-type, but not *TRPV4*^-/-^ rats, and suggested TRPV4 stimulation as a possible treatment of DU. In support of this, Takaoka et al. [109], using a similar rat DU model, demonstrated that intravesical application of GSK1016790A significantly decreased intercontraction intervals, bladder capacity, voided volume, and post void residuals without increasing NVCs, and that these effects were blocked by the TRPV4 antagonist RN1734.

### 8.2. TRP Antagonists

A large number of small-molecule TRP antagonists have been developed and their effects on LUT have been studied in normal animals and in different models of disease. Some of these drugs are briefly discussed below.

#### 8.2.1. TRPV1

Capsazepine is a competitive TRPV1 antagonist, but its low potency and lack of specificity in high concentrations necessitated development of new TRPV1 antagonists with potential applications also in the LUT [100,109,110,111,112,113,114,115]. A potent TRPV1 antagonist, GRC 6211 decreased bladder overactivity in a dose-dependent manner [113], when tested in two models of bladder inflammation, either acute, induced by acetic acid, or prolonged, induced by LPS [113]. The application of the same antagonist was also found to be effective in the reduction of bladder overactivity in chronic spinalized rats [114]. In low doses, GRC 6211 had no effect on bladder reflex activity of normal rats and wild type mice [113]. However, in high doses, it transiently blocked bladder contractions, and the effect was considered mediated via TRPV1, since the same dose did not produce any effect on TRPV1 KO mice. Another TRPV1 antagonist, JTS-653, blocked bladder afferent nerve firing and decreased bladder overactivity without affecting normal bladder function [115].

TRPV1 antagonists may cause hyperthermia following systemic administration, an effect well described in both animals [103] and humans [116,117,118,119,120]. In healthy subjects, Round et al. [116] studied the safety and pharmacokinetics of the TRPV1 antagonist, XEN-DO501, being developed for treatment of OAB. They found a dose-related increase in body temperature which attenuated over time and was not considered to be of clinical concern. Nash et al. [119] suggested that that TRPV1 antagonists with a classic polymodal inhibition profile could be identified where the analgesic action is separated from the effects on body temperature. In line with this, Brown et al. [120], studying the TRPV1 antagonist NEO6860 found no clinically significant increase in temperature or heat pain threshold/tolerance.

#### 8.2.2. TRPV4

It has been suggested that experimental novel TRPV4 antagonists can be expected to shed some light on the potential of TRPV4 inhibition for the treatment of inflammatory and neuropathic pain and various LUT disorders [100,121]. In mice and rats with experimental cystitis, HC-067047, a potent and selective TRPV4 antagonist increased functional bladder capacity and reduced micturition frequency [122]. Merrill and Vizzard [123] studied the functional role of TRPV4 in rats subjected to repeated variate stress (RVS). In these animals there were significant increases in TRPV4 transcript levels in urothelium but not detrusor smooth muscle. Bladder dysfunction, characterized by decreased bladder capacity and increased voiding frequency, was seen following RVS. In both controls and RVS animals, DO induced by intravesical administration of a TRPV4 agonist, GSK1016790A was improved by TRPV4 blockade using intravesical administration of HC067047. It was concluded that TRPV4 channel could be a promising target for bladder function disorders but so far information concerning effects of TRPV4 on human LUT does not seem to be available.

#### 8.2.3. TRPM8

The selective TRPM8 channel blocker, AMTB (N-(3-aminopropyl)-2-{[(3-methylphenyl) methyl]oxy}-N-(2-thienylmethyl)benzamide hydrochloride salt), was evaluated by Lashinger et al. [81]. In the anesthetized rat, intravenous administration of AMTB decreased the frequency of volume-induced bladder contractions, without reducing the amplitude of contraction. AMTB significantly attenuated reflex responses to noxious urinary bladder distension. The authors suggested that TRPM8 channel blockers can act on the bladder afferent pathway to attenuate the bladder micturition reflex and nociceptive reflex responses in the rat, and that targeting TRPM8 channel may provide a new therapeutic opportunity for DO/OAB and painful bladder syndromes.

Several new antagonists have been described [124,125], and some of them have been tested in animals for their effects on normal LUT and in models of LUT disorders [80,82,126,127,128,129,130].

## 9. Conclusions and Future Perspectives

In the LUT, several members of the TRP superfamily are involved in nociception and mechanosensory transduction. Animal studies have suggested a therapeutic potential of these channels, including TRPV1, TRPV2, TRPV4, TRM4, TRPM8 and TRPA1 for the treatment of both bladder over- and underactivity and bladder pain disorders; however, the translation of preclinical findings for clinical application has been slow. The therapeutic potential for TRPV1 channel desensitizing agonists (capsaicin, RTX) has been convincingly demonstrated. However, so far the potential of any of the channel blockers developed for non-bladder indications and tested in early human trials for safety has not been explored clinically in LUT dysfunction; and the adverse effect of hyperthermia of the first generation TRPV1 blockers has delayed development. Nevertheless, even if the transition from bench to bedside has been slow, TRP channels remain exciting targets for future LUT drugs.
